# Untargeted plasma proteomics nominates a novel set of plasma biomarkers for Alzheimer’s disease in African Americans

**DOI:** 10.1002/alz.086438

**Published:** 2025-01-09

**Authors:** Lindsey A Kuchenbecker, Joseph S. Reddy, Michael G. Heckman, John A. Lucas, Nilüfer Ertekin‐Taner, Neill R Graff‐Radford, Minerva M. Carrasquillo

**Affiliations:** ^1^ Mayo Clinic, Jacksonville, FL USA; ^2^ Department of Neurology, Mayo Clinic, Jacksonville, FL USA

## Abstract

**Background:**

Plasma biomarker development is of increasing interest due to improved accessibility compared to established diagnostic modalities for Alzheimer’s disease (AD). However, current plasma biomarker candidates have not been tested comprehensively in diverse populations, and these markers represent only a fraction of pathways perturbed in AD. Therefore, there is a need to identify novel plasma biomarkers that account for both disease heterogeneity and patient diversity. This study seeks to address these knowledge gaps through untargeted plasma biomarker discovery in African Americans (AA), an underrepresented group in AD research despite having a higher risk of developing AD compared to non‐Hispanic Whites.

**Methods:**

Untargeted proteomics were performed using the SomaScan 7k assay on plasma samples from AA study participants clinically diagnosed as AD (n=142) or cognitively unimpaired (CU, n=144). Linear regression models adjusted for age, sex, and batch were implemented to identify differentially expressed proteins (DEPs). APOE was additionally adjusted for in a sensitivity analysis. DEPs with the lowest q‐values were selected as predictors in a receiver operating characteristic (ROC) analysis to assess their accuracy to discriminate AD versus CU individuals.

**Results:**

A total of 153 DEPs were identified (q<0.05) when adjusting for age, sex, and batch, and 100 DEPs were identified when additionally adjusting for APOE. Of the 66 proteins overlapping between these models, SERPINA3, C7, IGFBP‐2, DKK3, SMOC1, and ACES had the lowest q‐values and were included in a ROC model adjusted for age, sex, and APOE. That model classified AD versus CU with an area under the curve (AUC) of 0.87 (95%CI: 0.83‐0.91), a 10.4% improvement over the base model including age, sex, and APOE (AUC=0.78; 95%CI: 0.72‐0.83). Despite the unbiased selection of proteins included in the ROC model, all selected proteins have been associated with AD and implicated in related functional pathways such as neuroinflammation, synaptic function, and insulin signaling.

**Conclusion:**

This preliminary plasma biomarker panel demonstrates the potential diagnostic capabilities of biomarker discovery through untargeted proteomics. Future directions include validation of findings in external datasets, concomitant assessment of p‐tau217, and evaluation of machine learning models to define the optimal diagnostic and predictive set of plasma protein biomarkers for AD in AA.

